# ZCCHC4 regulates esophageal cancer progression and cisplatin resistance through ROS/c-myc axis

**DOI:** 10.1038/s41598-025-89628-3

**Published:** 2025-02-12

**Authors:** Lihua Yao, Piao Wu, Fangyi Yao, Bo Huang, Fangmin Zhong, Xiaozhong Wang

**Affiliations:** 1 Department of Clinical Laboratory, Affiliated Hospital of North Sichuan Medical College, School of Clinical Medicine, North Sichuan Medical College, Nanchong, 637000 Sichuan China; 2 Jiangxi Province Key Laboratory of Immunology and Inflammation, Jiangxi Provincial Clinical Research Center for Laboratory Medicine, Department of Clinical Laboratory, The Second Affiliated Hospital, Jiangxi Medical College, , Nanchang University, Nanchang, 330006 China

**Keywords:** ZCCHC4, RNA-binding protein, Esophageal cancer, C-myc, Cisplatin resistance, Cancer, Computational biology and bioinformatics, Oncology

## Abstract

Zinc finger CCHC-type containing 4 (ZCCHC4) is a newly discovered *N*^6^-methyladenosine (m^6^A) RNA methyltransferase (MTase), which possesses an m^6^A MTase domain and an RNA-binding protein (RBP) Znf domain. Aberrantly expressed ZCCHC4 has been found to be correlated with poor prognosis and chemoresistance in various tumors, such as hepatocellular carcinoma, lung cancer and colorectal cancer. However, the expression and functional analysis of the role of ZCCHC4 in esophageal cancer (ESCA) is still elusive. The expression of ZCCHC4 in esophageal cancer tissues was evaluated by qPT-PCR and western blot. Serum esophageal tumor markers are detected by electrochemiluminescence immunoassay. Relationship between ZCCHC4 expression and pathway enrichment analysis were analyzed by R. The reactive oxygen species (ROS), cell proliferation, cell cycle and apoptosis of ZCCHC4 in esophageal squamous cell carcinoma (ESCC) cells tested by CCK8 assay and flow cytometry assay. Aberrant expression of ZCCHC4 is associated with cancer stages, lymph node metastasis (LNM), and tumor histology, and poorer Overall Survival (OS) in esophageal cancer. The mRNA level of ZCCHC4 in esophageal cancer patients correlates with serum carcinoembryonic antigen (CEA) levels, Squamous Cell Carcinoma (SCC) markers, and tissue polypeptide antigen (TPA) levels. Knockdown of ZCCHC4 induces DNA damage, leading to an elevation of reactive oxygen species (ROS), which in turn triggers S-phase arrest, enhances apoptosis, augments sensitivity to cisplatin treatment, and inhibits proliferation in esophageal cancer cells. Conversely, overexpression of ZCCHC4 promotes proliferation, inhibits apoptosis, and increases resistance to cisplatin in esophageal cancer cells. Furthermore, scavenging ROS reverses the effects of ZCCHC4 downregulation on both proliferation and apoptosis in esophageal cancer cells. Additionally, downregulation of ZCCHC4 inhibits the progression of esophageal cancer and reduces cisplatin resistance in vivo. In summary, downregulation of ZCCHC4 leads to increased sensitivity of ESCC cells to cisplatin, inhibits proliferation, and promotes apoptosis in esophageal cancer cells, potentially via the ROS/c-myc axis. The study suggests a potential adjunctive role for ZCCHC4 in the diagnosis and treatment of esophageal cancer and aids in further understanding the underlying mechanisms in ESCA progression.

**Supplementary Information:**

The online version contains supplementary material available at 10.1038/s41598-025-89628-3.

## Introduction

Esophageal cancer is the sixth leading cause of cancer-related death worldwide and is characterized by its early non-diagnosis and frequent metastasis^1^. ESCC is the dominant histological subtype of esophageal cancer, accounting for approximately 90% of newly diagnosed cases in China^2^. Early-stage ESCC often carries a favorable prognosis, while advanced disease is associated with a poor prognosis^3^. Therefore, exploring the mechanisms underlying the occurrence and development of esophageal cancer is very helpful for its diagnosis and treatment.

Zinc finger CCHC-type containing 4 (ZCCHC4), also known as HSPC052/ZGRF4, is an RNA-binding protein (RBP) Znf domain^4^. RBPs play significant roles in cancer progression and treatment^5,]^^6,]^^7^. According to recent reports, ZCCHC4 is involved in tumorigenesis. Ma et al. revealed that downregulating ZCCHC4 reduces the proliferation of hepatocellular carcinoma (HCC) cell lines and the growth of xenogeneic tumors in mice^8^. Meanwhile, upregulated ZCCHC4 expression was found to be associated with poor prognosis in liver cancer patients and to promote tumor growth and chemoresistance of HCC cells^9^. In small cell lung cancer, ZCCHC4 was found to be correlated with worse overall survival (OS) and chemotherapy resistance^10^. However, the expression and function of ZCCHC4 in ESCA have yet to be identified.

In this study, we investigated the expression of ZCCHC4 mRNA and protein, which was higher in cancer tissues and significantly associated with cancer stages, nodal metastasis status, and tumor markers. Furthermore, downregulation of ZCCHC4 increases sensitivity to cisplatin treatment, promotes apoptosis, and inhibits proliferation of esophageal cancer cells, potentially through the ROS/c-myc axis. This helps us further understand the underlying mechanisms in ESCA progression.

## Materials and methods

### Data processing and ZCCHC4 expression analysis

ZCCHC4 expression in pan-cancer was also analyzed in TIMER 2.0 (http://timer.cistr-ome.org/*)* using TCGA data^11^. ZCCHC4 protein expression in pan-cancer was analyzed in the Human Protein Atlas.

### Populations

ESCC tissues and paracarcinoma normal tissues samples were surgically isolated within 30 min to soaked in RNAlater solution (Ambion, Carlsbad, CA, USA), and stored at -80 °C until further processing. This study was approved by the Medical Ethics Committee of the Second Affiliated Hospital of Nanchang University. Each participant provided a written informed consent for participation. All methods were performed in accordance with the relevant guidelines and regulations.

### RNA extraction and quantitative real-time PCR

The total RNA was extracted from tissues and PBMCs using the TRIzol reagent (Ambion, Carlsbad, CA, USA) according to the manufacturer’s instructions. ZCCHC4 forward 5’-CCCGTGCGTATTTTCACCAA-3’, ZCCHC4 reverse 5’-GGTTCCATTTCCTGCCATCC-3’, c-myc forward 5’- AAGGTCAGAGTCTGGATCAC-3’, c-myc reverse 5’- TAACTACCTTGGGGGCCTTT-3’, and β-actin forward 5’-GGACTTCGAGCAAGAGATGG-3’ and β-actin reverse 5’-AGCACTGTGTTGGCGTACAG-3’.

### Western blotting (WB) assay

WB assays were performed to measure ZCCHC4 protein expression in tissues and cells, as previously described^12^. Primary antibodies against anti-ZCCHC4 (1:1000), c-myc (1:1000), p-H2A.X (1:1000) and β-actin (1:1000) were obtained from Cell Signaling Technology (CST; Danvers, MA, USA).

### Functional and pathway enrichment analysis

The relevance of ZCCHC4 to hallmark sets in ESCA were investigated by the “Gene set variation analysis” (GSVA) package.

### Cell Culture and transfections

This work cultivated ESCA cells (KYSE150, ECA10) were obtained from the Cell Bank of the Shanghai Institute of Cell Biology (Chinese Academy of Medical Sciences, Shanghai, China) and maintained in RPMI-1640 medium (Gibco, Grand Island, NY, USA) containing 1% penicillin/streptomycin (P/S; Invitrogen, Waltham, MA, USA) and 10% fetal bovine serum (FBS, Gibco) at 37 °C and 5% CO_2_. Cells were transfected with shRNA designed and synthesized by GenePharm (Shanghai, China), overexpression of ZCCHC4 was performed using expression plasmid, puromycin was added to screen for stable cell lines. The transfection efficiency was evaluated by qRT-PCR and western blot.

### Cell proliferation assay and flow cytometry assay

The CCK-8 assay was performed to measure cell viability as previously described^13^. The assay was performed in triplicate. The transfected cells were resuspended in 70% ethanol for 2 h. After centrifugation, the cell pellets were resuspended in PI solution and analyzed cell cycle using flow cytometry. The transfected cells were washed with PBS and stained with annexin V-APC and propidium lodide at room temperature in dark for 30 min, then cell apoptosis was detected by flow cytometry. The intracellular ROS levels was conducted with the ROS Assay Kit (Thermo Fisher).

### Animal studies

The study is reported in accordance with ARRIVE guidelines. The animal experiment was approved by the Experimental Animal Center of Nanchang University. All methods were performed in accordance with the relevant guidelines and regulations. BALB/c nude mice, approximately 4 weeks of age, were obtained from GemPharmatech (Nanjing, China). For the in vivo tumor growth assay, the mice were subcutaneously injected with stably transfected KYSE150 cells (5 × 10^6^). Tumor volumes were measured every 3 days starting from the 7th day. At 28 days, the tumors were removed and weighed. In the cisplatin-treated group, mice were intraperitoneally injected with cisplatin (3 mg/kg every 2 days) from day 7 until the end of the experiment.

### Statistical analysis

R software (4.2.1) and R packages were applied to the statistical analyses. The Wilcoxon rank-sum test was performed to analyze distinctions between two groups, and correlation coefficients were calculated using Spearman’s correlation analysis. Statistical significance was set at two-sided p-values < 0.05. GraphPad Prism 9.0 (GraphPad, San Diego, CA, USA) were used for the data analysis. Statistical significance was set at *p* < 0.05.

## Results

### ZCCHC4 expression and clinicopathological features in esophageal cancer patients

**Fig. 1:**
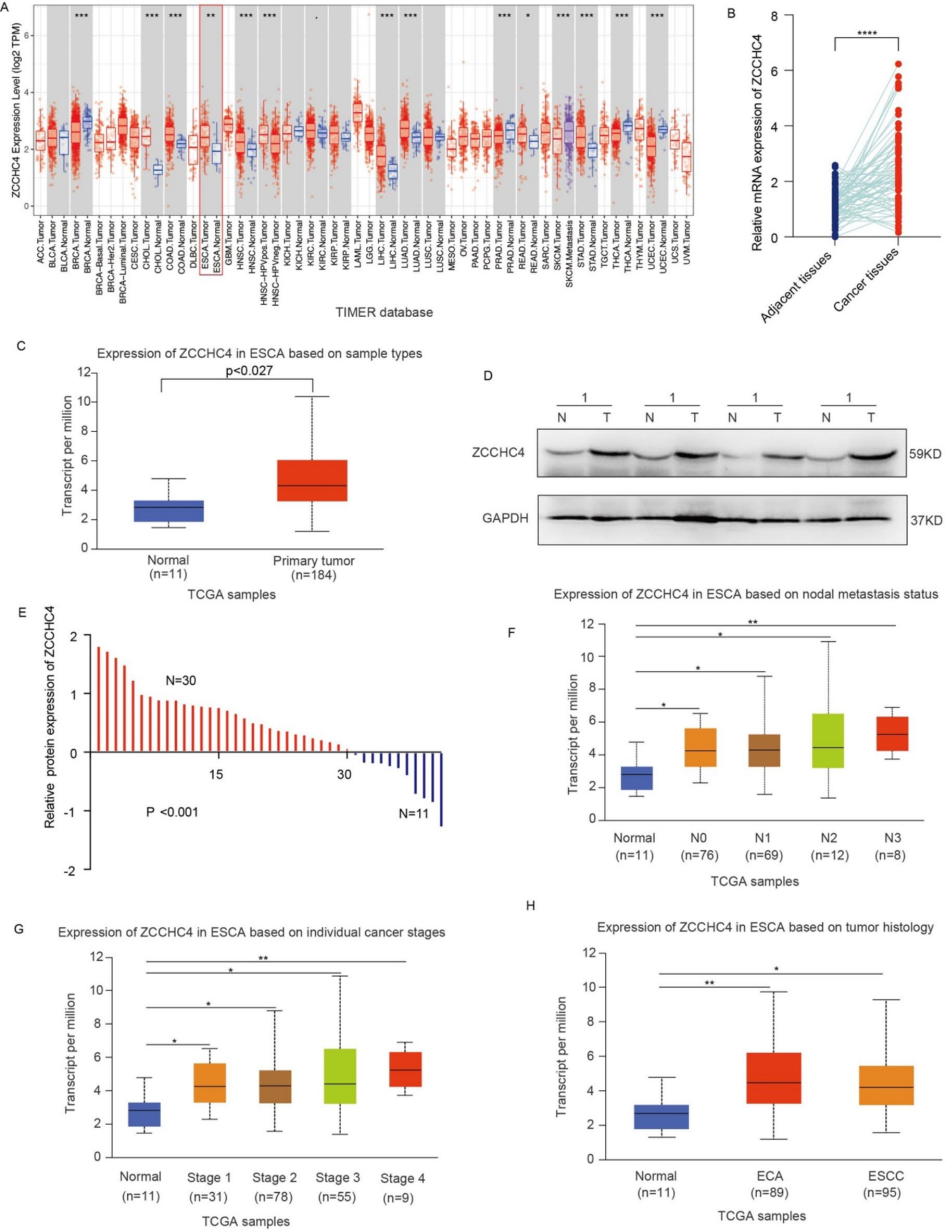
ZCCHC4 expression and clinicopathological features in esophageal cancer patients. (**A**) The mRNA expression of ZCCHC4 in TIMER database. (**B**) The mRNA expression of ZCCHC4 in ESCC patients. (**C**) The transcription level of ZCCHC4 in TCGA database. (**D**-**E**) The ZCCHC4 protein levels among 41 ESCC tissues. the original blots are presented in Fig. S1.(**F**-**H**) The correlation of ZCCHC4 transcriptional expression and lymph node metastasis statuses, cancer stages 1–4 and tumor histology. *p < 0.05; **p < 0.01; ***p < 0.001.

The transcriptional expression of ZCCHC4 in different cancers was explored using the TIMER database (Fig. 1A), ZCCHC4 mRNA expression was significantly upregulated in ESCA patients. To clarify the abnormal expression of ZCCHC4 in esophageal cancer, the ZCCHC4 mRNA level of ESCC tumors and adjacent non-tumor tissues was detected by qRT-PCR (Fig. 1B), which proved that tumor tissues were significantly higher than the control group, and this result is consistent with the results in the TCGA database (Fig. 1C). Meanwhile, we detected the ZCCHC4 protein levels in 41 pairs of ESCC tissues, and 30 cases of tumor tissues showed significantly over-expression (Fig. 1D-E). Next, the relationship between ZCCHC4 levels and clinical pathological characteristics is explored, higher mRNA level of ZCCHC4 was significantly associated with lymph node metastasis statuses N0, N2, N3, and N4 (Fig. 1F), cancer stages 1–4 (Fig. 1G) and tumor histology (Fig. 1H). Interestingly, the analysis of the pathological data we collected also demonstrated that the expression of ZCCHC4 is related to TNM stages and Lymph node metastasis (Table 1). These results demonstrated the transcription and protein levels of ZCCHC4 are abnormally expressed in esophageal cancer, and ZCCHC4 mRNA expression significantly associated with clinicopathological characteristics.

**Table 1:** Association of clinicopathological data and expression of ZCCHC4 in ESCC patients.

Patient characteristics	ZCCHC4 expression		X^2^	*p*-value
	High (*n* = 47)	Low (*n* = 23)		
Age			2.664	0.103
< 65	24	7		
≥ 65	23	16		
Gender			0.024	0.876
Male	36	18		
Female	11	5		
Tumor size			1.157	0.561
< 3 cm	9	7		
3–5 cm	35	15		
> 5 cm	3	1		
Tumor location			4.104	0.129
Upper	4	6		
Middle	33	14		
Lower	10	3		
TNM stage			4.622	0.032*
I–II	9	10		
III–IV	38	13		
Lymph node metastasis			5.245	0.022*
Yes	28	7		
NO	19	16		
Differention			2.387	0.303
Well	14	8		
Moderate	19	12		
Poor	14	3		

Using a Fisher’s exact test. The *p*-value was set at 0.05 and * indicates *p* < 0.05.

### Correlations between ZCCHC4 level and tumor markers among esophageal cancer patients

**Fig. 2:**
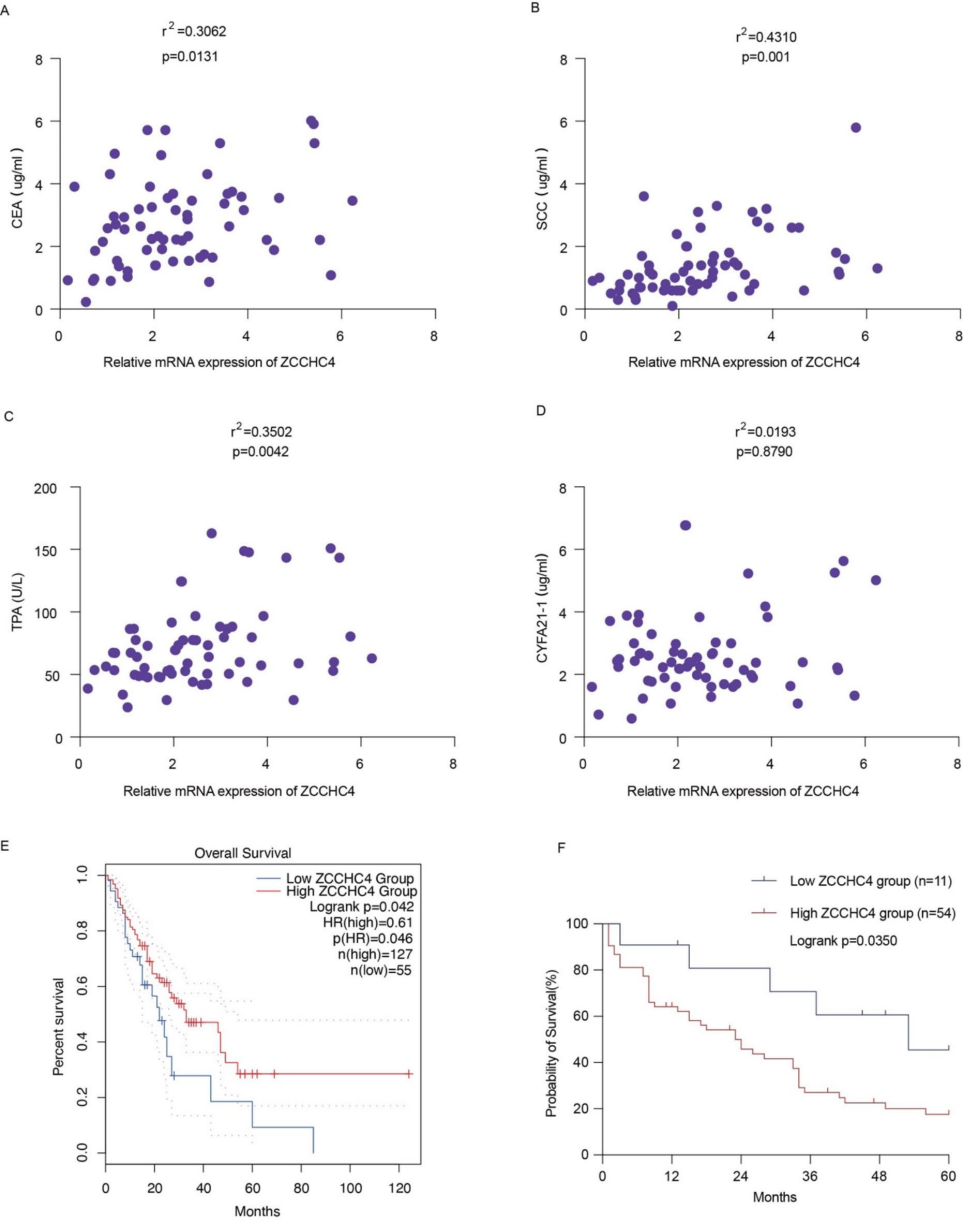
Correlation between ZCCHC4 expression and esophageal tumor markers. (**A**-**D**). The mRNA expression of ZCCHC4 correlated with the serum level of CEA, SCC, CYFRA21-1, and TPA. (**E**). The prognostic significance of ZCCHC4 in esophageal cancer in the GEPIA database. (**F**) KM survival analysis of comparing two subgroups with high and low expression of ZCCHC4 in 65 esophageal cancer patients. *p < 0.05; **p < 0.01; ***p < 0.001.

Esophageal tumor markers (CEA, SCC, TPA, CYFRA21-1) emerge as multifaceted biomarkers, not merely confined to their role as auxiliary diagnostic aids in esophageal cancer but also intimately intertwined with the prognosis of patients, as evidenced by the dynamic fluctuations in their serum concentrations. Therefore, we further analyzed the correlation between the expression level of ZCCHC4 and tumor markers, the findings presented a compelling correlation between the mRNA expression levels of ZCCHC4 and a panel of tumor biomarkers, including CEA, SCC, TPA (Fig. 2A-C). However, there is no significant association between ZCCHC4 expression and CYFA21-1 levels (Fig. 2D). Next, Fig. 2E-F revealed that the group with a higher expression level of ZCCHC4 had the worse OS rate. The present findings offer compelling evidence that ZCCHC4 emerges as a promising candidate biomarker, complementary to established tumor markers, and may contribute to a more comprehensive molecular profiling strategy for ESCC diagnosis and prognosis.

### Correlation between ZCCHC4 and c-myc targets pathway

**Fig. 3:**
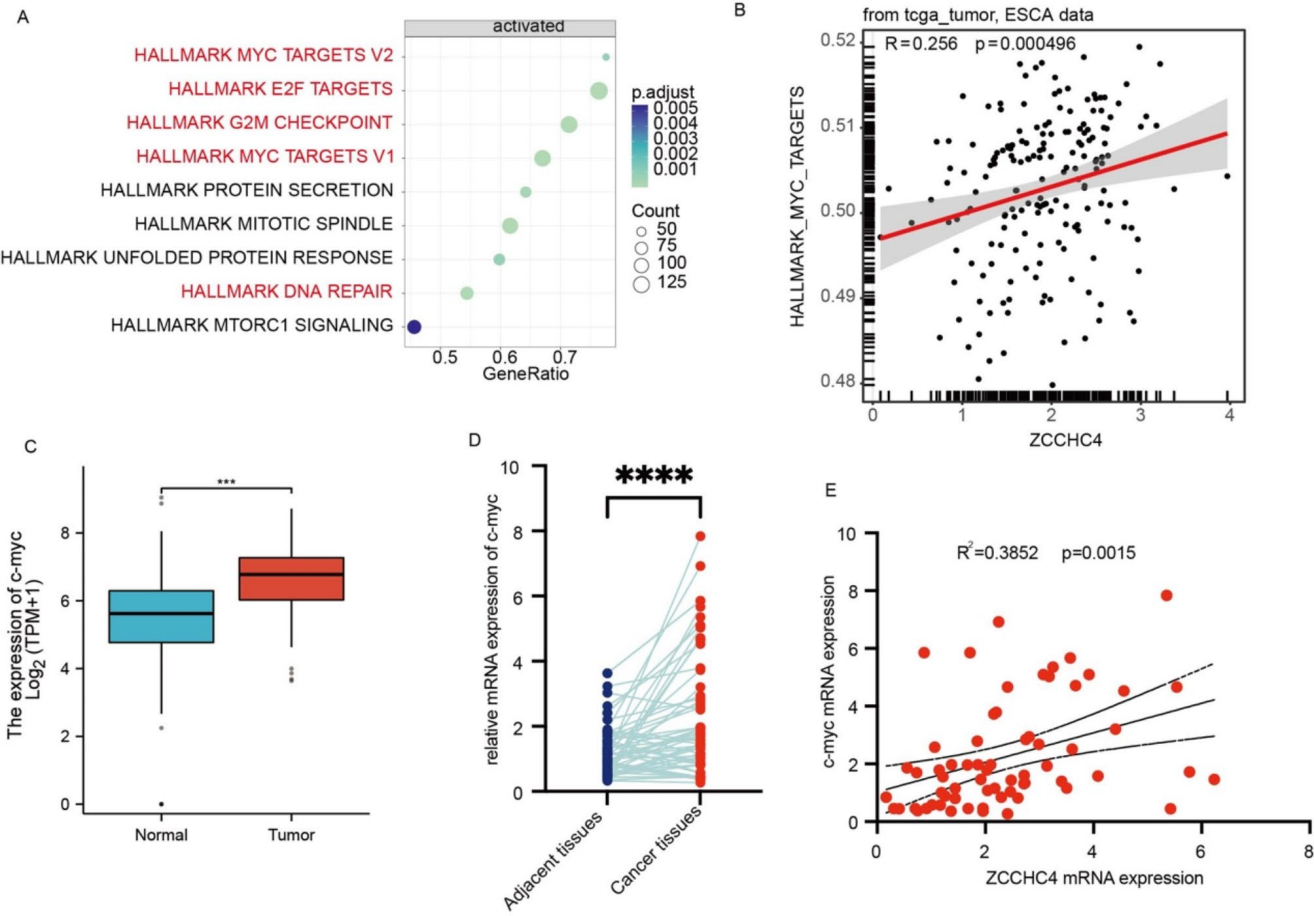
Correlation between ZCCHC4 and c-myc targets pathway. (**A**) Bar plots demonstrated correlations between ZCCHC4 and hallmark sets in ESCA. (**B**) ZCCHC4 expression correlated to hallmark MYC targets. (**C**) The mRNA expression of ZCCHC4 and MYC in TCGA database. (**D**) The mRNA expression ZCCHC4 and MYC in ESCC tissues. (**E**) Correlation between ZCCHC4 and MYC mRNA expression in ESCC tissues. *p < 0.05; **p < 0.01; ***p < 0.001.

To further explore the functional roles of ZCCHC4 in esophageal carcinoma, we employed the “Gene Set Variation Analysis” (GSVA) package to investigate the relevance of ZCCHC4 to hallmark sets within the context of ESCA. The Bubble diagram revealed ZCCHC4 was strongly associated with pathways of cell proliferation, including c-myc targets, E2F targets, G2M checkpoints, DNA repair, and mTOR signaling in ESCA (Fig. 3A), the c-myc targets pathway emerges as the most prominent enriched pathway. Notably, a marked correlation is observed between the c-myc targets pathway and ZCCHC4 (Fig. 3B). Furthermore, we examined the expression of the oncogenic c-myc in esophageal cancer tissues, our findings reveal a marked upregulation of c-myc compared to adjacent non-tumor tissues (Fig. 3D). Specifically, a positive correlation was observed between c-myc’s mRNA expression and ZCCHC4 levels (Fig. 3E), corroborating the results derived from TCGA database analysis (Fig. 3C). This concordance strengthens the hypothesis of a functional link between ZCCHC4 and the c-myc pathway in esophageal cancer.

### Downregulation of ZCCHC4 suppresses proliferation, promotes apoptosis and increases cisplatin sensitivity of esophageal cancer cells

**Fig. 4:**
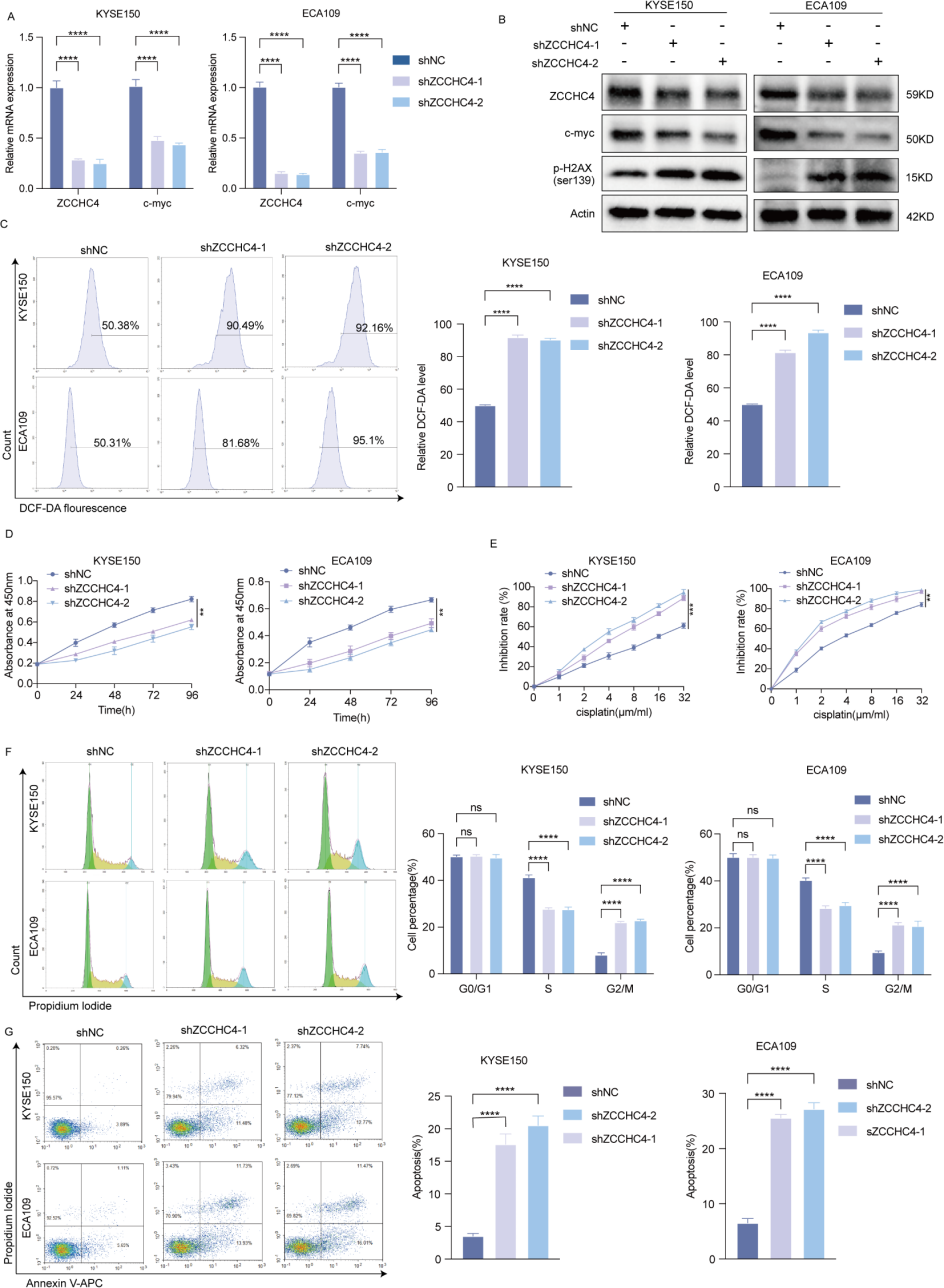
Downregulation of ZCCHC4 suppresses proliferation, promotes apoptosis and increases cisplatin sensitivity of esophageal cancer cells. (**A**) The mRNA expression of ZCCHC4 and c-myc detected by qRT-PCR. (**B**) The protein abundance of ZCCHC4, c-myc and p-H2A.X was determined using WB, the samples derive from the same experiment and blots were processed in parallel and the original blots are presented in Fig. S1. (**C**) The levels of ROS were examined by flow cytometry assay. (**D**) The cell viability was assessed by CCK8 assay. (**E**) The inhibition rate of esophageal cancer cells treated with different concentrations of cisplatin was detected by CCK-8 assay. (**F**-**G**) The cell cycle and apoptosis were examined by flow cytometry assay. *p < 0.05; **p < 0.01; ***p < 0.001; ****p < 0.0001.

To further delve into the functional roles of ZCCHC4 in esophageal cancer, we used shRNA to knockdown ZCCHC4 and found that the expression of c-myc was also decreased, while the DNA damage marker p-H2A.X was significantly increased (Fig. 4A-B). DNA damage induces the generation of ROS^14^, and elevated levels of reactive oxygen species (ROS) can impede tumor progression by suppressing c-myc^15^, leading us to hypothesize that ZCCHC4 may regulate c-myc expression downregulation via ROS modulation. Flow cytometry analysis showed an augmentation in ROS production in esophageal cancer cells with ZCCHC4 knockdown (Fig. 4C). When ZCCHC4 was depleted, the viability of ESCC cells was significantly inhibited (Fig. 4D), the sensitivity of ESCC cells to cisplatin has increased (Fig. 4E), cell cycle arrest in the G2 phase and a reduction in the S phase (Fig. 4F) and apoptosis was markedly increased (Fig. 4G). These results suggest that downregulation of ZCCHC4 suppress esophageal cancer cell proliferation, potentially through the production of ROS.

### Scavenging ROS can rescue the proliferation and reduce apoptosis in ZCCHC4-knockdown esophageal cancer cells

**Fig. 5:**
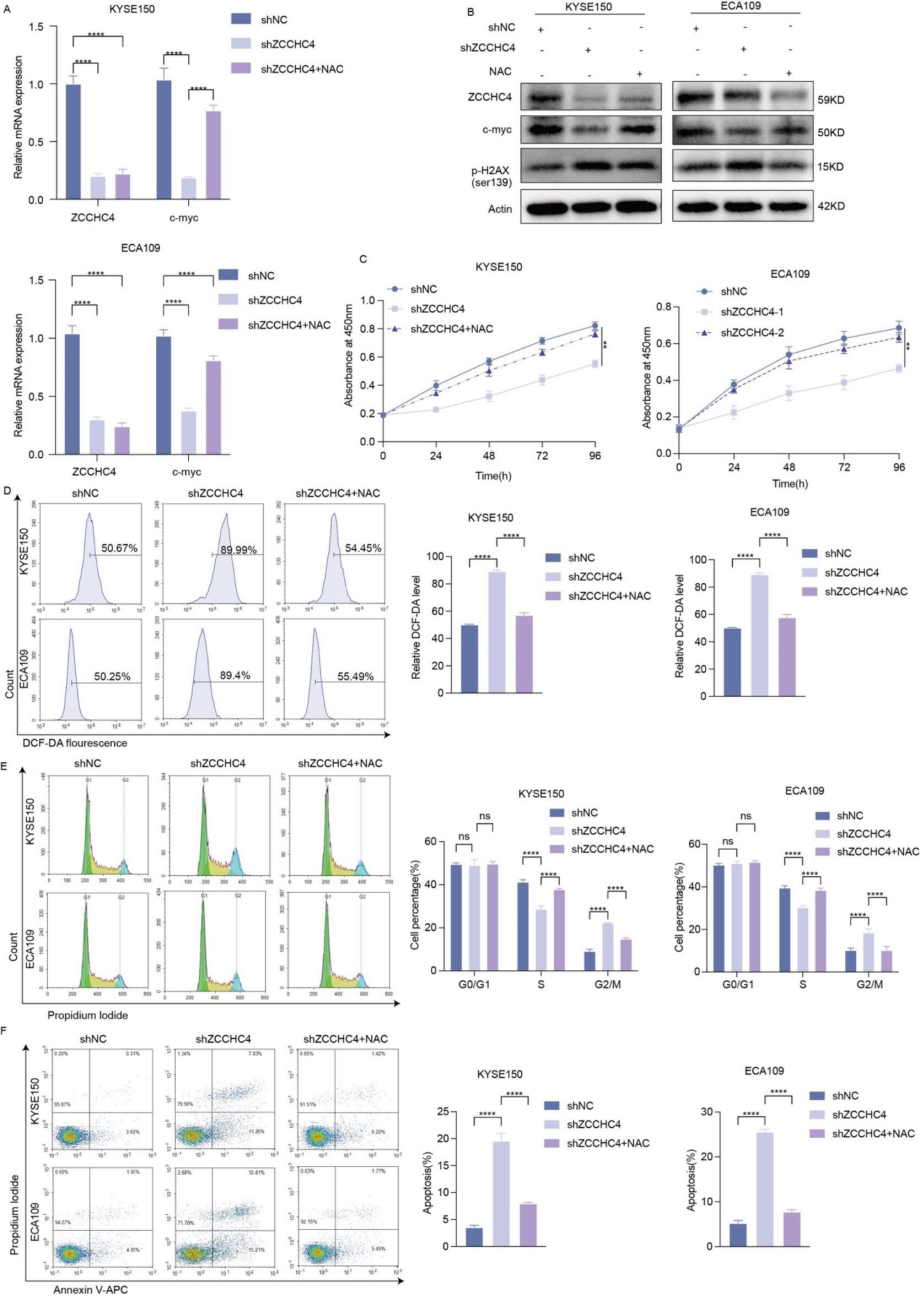
Scavenging ROS can rescue the proliferation and reduce apoptosis in ZCCHC4-knockdown esophageal cancer cells. (**A**) The mRNA expression of ZCCHC4 and c-myc detected by qRT-PCR. (**B**) The protein abundance of ZCCHC4, c-myc and p-H2A.X was determined using WB, the samples derive from the same experiment and blots were processed in parallel and the original blots are presented in Fig. S1. (**C**) The cell viability was assessed by CCK8 assay. (**D**-**F**) The levels of ROS, cell cycle, and apoptosis were examined by flow cytometry assay. *p < 0.05; **p < 0.01; ***p < 0.001; ****p < 0.0001.

To validate whether ZCCHC4 knockdown inhibits c-myc through highly reactive oxygen species (ROS), thereby modulating the proliferation of esophageal cancer cells, N-acetylcysteine (NAC) effectively reduced the elevated levels of ROS that were observed following the knockdown of ZCCHC4 (Fig. 5D). Consequently, the downregulation of c-myc expression elicited by shZCCHC4 was partially reversed, concurrent with a decrease in the expression of p-H2A.X (Fig. 5A-B). Furthermore, the suppressed cell viability observed in cells transfected with shZCCHC4 was rescued (Fig. 5C), accompanied by an increase in the S phase of the cell cycle (Fig. 5E) and a decrease in apoptosis (Fig. 5F). In summary, scavenging ROS reversed the effect of ZCCHC4 knockdown on ESCC cell proliferation and apoptosis.

### Overexpression of ZCCHC4 promotes proliferation, inhibits apoptosis, and increases cisplatin resistance in esophageal cancer cells

**Fig. 6:**
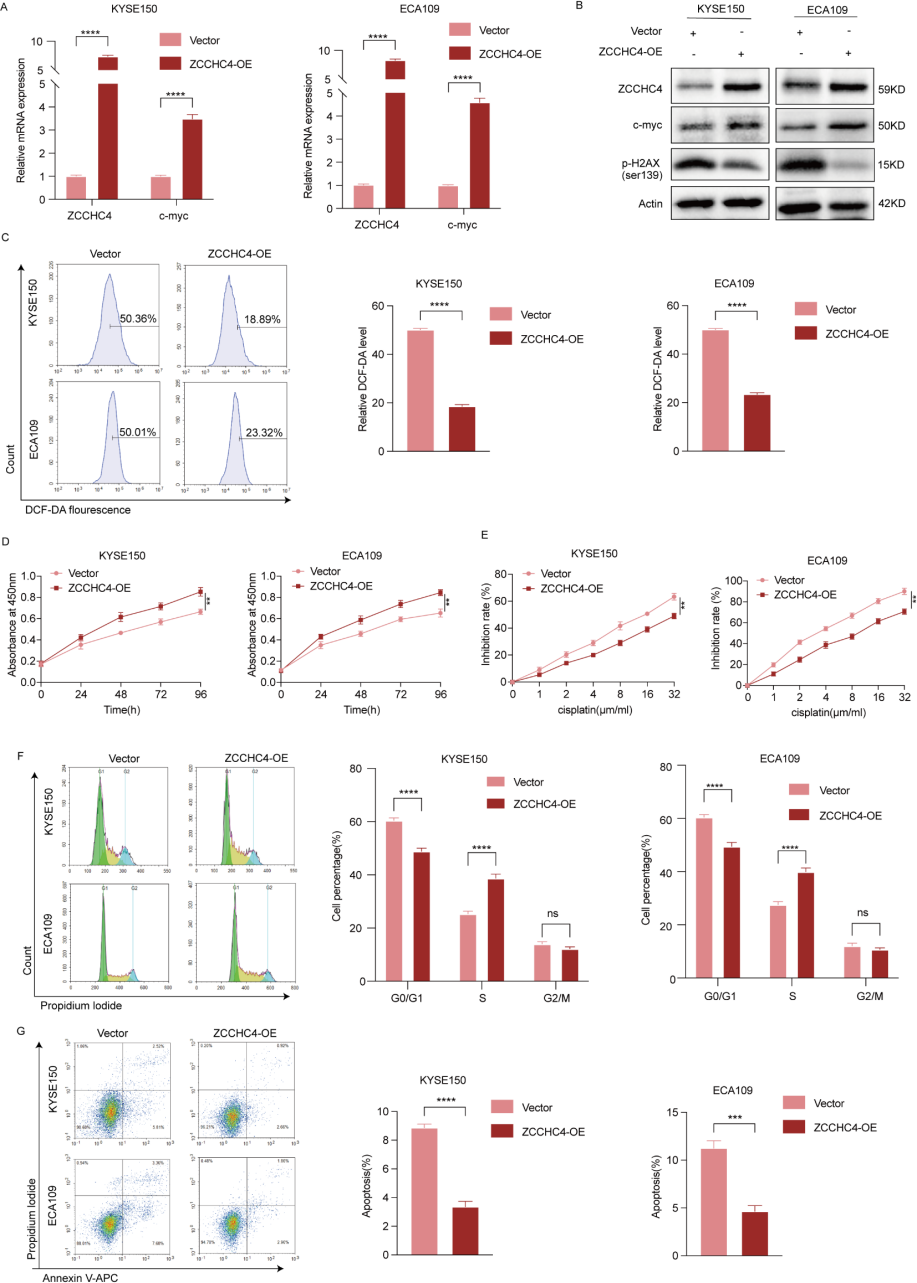
Overexpression of ZCCHC4 promotes proliferation, inhibits apoptosis, and increases cisplatin resistance in esophageal cancer cells. (**A**) The mRNA expression of ZCCHC4 and c-myc detected by qRT-PCR. (**B**) The protein abundance of ZCCHC4, c-myc and p-H2A.X was determined using WB, the samples derive from the same experiment and blots were processed in parallel and the original blots are presented in Fig. S1. (**C**) The levels of ROS were examined by flow cytometry assay. (**D**) The cell viability was assessed by CCK8 assay. (**E**) The inhibition rate of esophageal cancer cells treated with different concentrations of cisplatin was detected by CCK-8 assay. (**F**-**G**) The cell cycle and apoptosis were examined by flow cytometry assay. *p < 0.05; **p < 0.01; ***p < 0.001; ****p < 0.0001.

Conversely, in the ZCCHC4 over-expressing cell lines, the expression of c-myc increased and p-H2AX decreased (Fig. 6A-B), the level of ROS was decreased (Fig. 6C). ZCCHC4 over-expression promoted the proliferation (Fig. 6D) of ESCC cells and increased cisplatin resistance (Fig. 6E). Next, we employed flow cytometry to investigate the effects of ZCCHC4 overexpression on cell cycle progression and apoptosis in ESCC cells, compared with the control group, the G0/G1 phase of the cell cycle was reduced (Fig. 6F), whereas the S phase was increased, and apoptosis was decreased (Fig. 6G) in the ZCCHC4-overexpressing group. The results suggested ZCCHC4 promotes proliferation, inhibits apoptosis, and increases cisplatin resistance in esophageal cancer cells.

### Knockdown of ZCCHC4 inhibits esophageal cancer progression and reduces cisplatin resistance in vivo

**Fig. 7:**
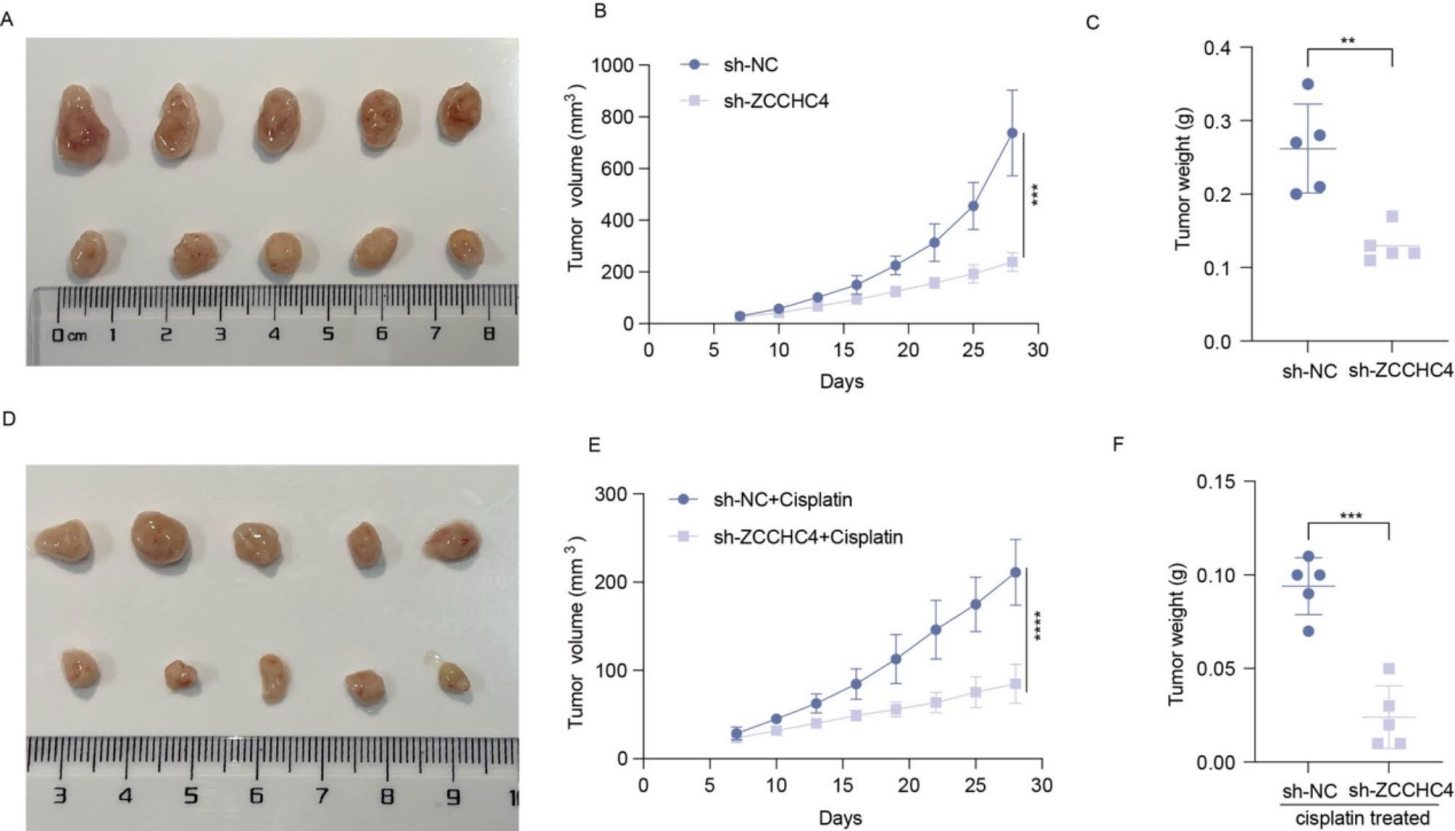
Knockdown of ZCCHC4 inhibits esophageal cancer progression and reduces cisplatin resistance in vivo. (**A**-**C**) Representative tumor images (**A**), tumor volume growth curves (**B**) and tumor weight (**C**) in ZCCHC4 knockdown group and control group. (**D**-**F**) Representative tumor images (**D**), tumor volume growth curves (**E**) and tumor weight (**F**) in ZCCHC4 knockdown group and control group after treated with cisplatin. *p < 0.05; **p < 0.01; ***p < 0.001; ****p < 0.0001.

Since our findings indicate that ZCCHC4 promotes the proliferation of esophageal cancer cells and enhances cisplatin resistance, we investigated whether knockdown of ZCCHC4 inhibits the progression of esophageal cancer and increases sensitivity to cisplatin in vivo. First, the final volume of subcutaneous tumors in the ZCCHC4 knockdown group was significantly smaller than that of the control group (Fig. 7A). In observing the trends in tumor growth, it is evident that the subcutaneous tumor growth in the knockdown group is markedly suppressed and slowed (Fig. 7B). Furthermore, when comparing the tumor weights between the two groups, the results demonstrate a significant reduction in tumor weight in the knockdown group (Fig. 7C). Consistent with in vitro findings, mice with subcutaneous tumors derived from ESCC cells in which ZCCHC4 was knocked down exhibited significantly inhibited tumor growth (Fig. 7D), as well as reduced tumor volume (Fig. 7E) and weight (Fig. 7F) following treatment with cisplatin. In summary, we demonstrate that knockdown of ZCCHC4 inhibits esophageal cancer progression and reduces cisplatin resistance in vivo.

## Discussion

Esophageal cancer, a malignancy characterized by its propensity for late-stage diagnosis, heightened metastatic capacity^16^. Currently, the therapeutic armamentarium for esophageal cancer predominantly comprises surgical resection, chemotherapy, radiation therapy, and molecular targeted therapies^17,18^. However, the prognosis and overall outcomes of esophageal cancer patients remain bleak^19^. Consequently, there is a pressing need to delve into the identification of early diagnostic biomarkers, as well as the discovery of novel therapeutic targets for esophageal cancer.

ZCCHC4 is a newly identified m6A regulator that is abnormally expressed in tumors and closely related to proliferation, chemoresistance, and prognosis of malignancies^8–10^. To the best of our knowledge, this is the first study reporting on ZCCHC4 in ESCA. The present study is aim to explore aberrant expression, diagnosis, prognosis, and function of ZCCHC4 in ESCA.

Previous studies have suggested that high ZCCHC4 levels are associated with liver cancer, colon cancer, small cell lung cancer^9,10,20^. The expression of ZCCHC4 frequently exhibit an upward trend in neoplastic tissues, albeit with instances of down-regulation observed in select tumors, suggesting a tissue-specific functionality. Consequently, elucidating the intricate expression landscape of ZCCHC4 specifically within ESCA serves as an indispensable prerequisite for unraveling its potential role in ESCA. Analyses of databases, coupled with our findings from ESCA tissue samples, consistently indicate an elevated expression of ZCCHC4 in ESCA. Notably, this heightened expression of ZCCHC4 is significantly correlated with TNM stages and lymph node metastasis, underscoring its potential clinical relevance. Furthermore, the mRNA levels of ZCCHC4 exhibit an association with serum esophageal tumor markers in patients, hinting at its potential utility in diagnostic and prognostic contexts. Moreover, higher expression level of ZCCHC4 had the poorer OS rate in esophageal cancer patients. Collectively, these results suggest that ZCCHC4 is aberrantly overexpressed in ESCA and may play a pivotal role in patient diagnosis and prognosis. However, the potential of ZCCHC4 as a diagnostic biomarker for tumors, as well as the prognostic value of changes in ZCCHC4 levels before and after treatment in assessing therapeutic efficacy and prognosis, necessitates further research to explore the sensitivity and specificity of diagnosis. Additionally, the convenience of detection methods poses a significant challenge that warrants attention.

In pathway analysis, ZCCHC4 was positively correlated with the genes related to c-myc targets, the G2M checkpoint, E2F targets, protein secretion, and DNA repair in ESCA. Recent studies demonstrated that ZCCHC4 affected cell growth in cancers^8,21^, especially in gastrointestinal tumors^9,20^. In HCC cells, ZCCHC4 could regulate the DNA-damage response (DDR) and inhibit DDR-related signals, and p-ATM signaling is an important PI3K kinase^9^. In our study, knockdown of ZCCHC4 induces DNA damage, DNA damage induces the generation of reactive oxygen species^14^. However, the intricate relationship between ZCCHC4 and the c-myc targets remains largely unexplored and uncharacterized. ROS production and ROS limitation pathways are common features of cancer cells^22,23^. Moderate levels of ROS can cause oxidative stress that can inflict damage upon vital cellular components, including proteins, DNA, and lipids, while excessive concentrations of ROS can initiate a programmed cell death pathway in cancer, such as apoptosis and ferroptosis^24,25^. The c-myc proto-oncogenes occupy a prominent position among the most frequently activated oncoproteins in the landscape of human neoplasia, serving as pivotal regulators that orchestrate diverse cellular programs^26,27,]^^28,29^. Previous research has established that the expression of c-myc is upregulated in esophageal cancer^30,31^, findings that are consistent with our study. Several studies have reported the involvement of reactive oxygen species (ROS) in the regulation of myc^15,32,33^. In our investigation, knockdown of ZCCHC4 induces ROS accumulated, downregulated c-myc and promoted DNA damage. Next, downregulation of ZCCHC4 leads to increased sensitivity of ESCC cells to cisplatin, suppression of cell viability, a reduction in the S phase of the cell cycle, and the induction of apoptosis in esophageal cancer cells. Conversely, ZCCHC4 over-expression promotes proliferation, inhibits apoptosis, and increases cisplatin resistance in esophageal cancer cells. To further investigate the regulation of ZCCHC4 on the proliferation of ESCC cells via ROS/c-myc axis. The ROS scavengers (NAC) was able to rescue the downregulation of c-myc expression induced by ZCCHC4 knockdown. Furthermore, NAC treatment reversed the inhibition of cellular viability in ZCCHC4-konckdown cells and reduced the onset of apoptosis. These findings underscore the pivotal role of ZCCHC4 in regulating ROS homeostasis and c-myc-mediated signaling, thereby modulating ESCC cell proliferation and apoptosis. From a functional perspective, we demonstrated that downregulation of ZCCHC4 inhibits proliferation, promotes apoptosis in esophageal cancer, and also enhancing sensitivity to cisplatin treatment. Consequently, further identification of specific ZCCHC4 inhibitors holds direct translational significance for the treatment of esophageal cancer in the future.

In summary, the present study elucidates the overexpression of ZCCHC4 in esophageal cancer, with a marked correlation observed between ZCCHC4 mRNA levels and clinicopathological features, suggesting a potential adjunctive role for ZCCHC4 in the diagnosis and prognosis of esophageal cancer. Downregulation of ZCCHC4 leads to increased sensitivity of ESCC cells to cisplatin, and inhibits the proliferation and promotes apoptosis of esophageal cancer cells, potentially via the ROS/c-myc axis. However, our research has several limitations, we did not demonstrate the direct binding mechanism between ZCCHC4 and c-myc, the precise underlying mechanisms necessitate further investigation in the future.

## Electronic supplementary material

Below is the link to the electronic supplementary material.

Supplementary Material 1

## Data Availability

The datasets used for the current study are available from online websites. All data generated or analyzed during this study are included in this published article and its supplementary information files.
