# Supplementary figures and images for: ZCCHC4 regulates esophageal cancer progression and cisplatin resistance through ROS/c-myc axis
Source: Sci Rep. 2025 Feb 12;15:5149. doi: 10.1038/s41598-025-89628-3 (PMC11814405; doi:10.1038/s41598-025-89628-3)

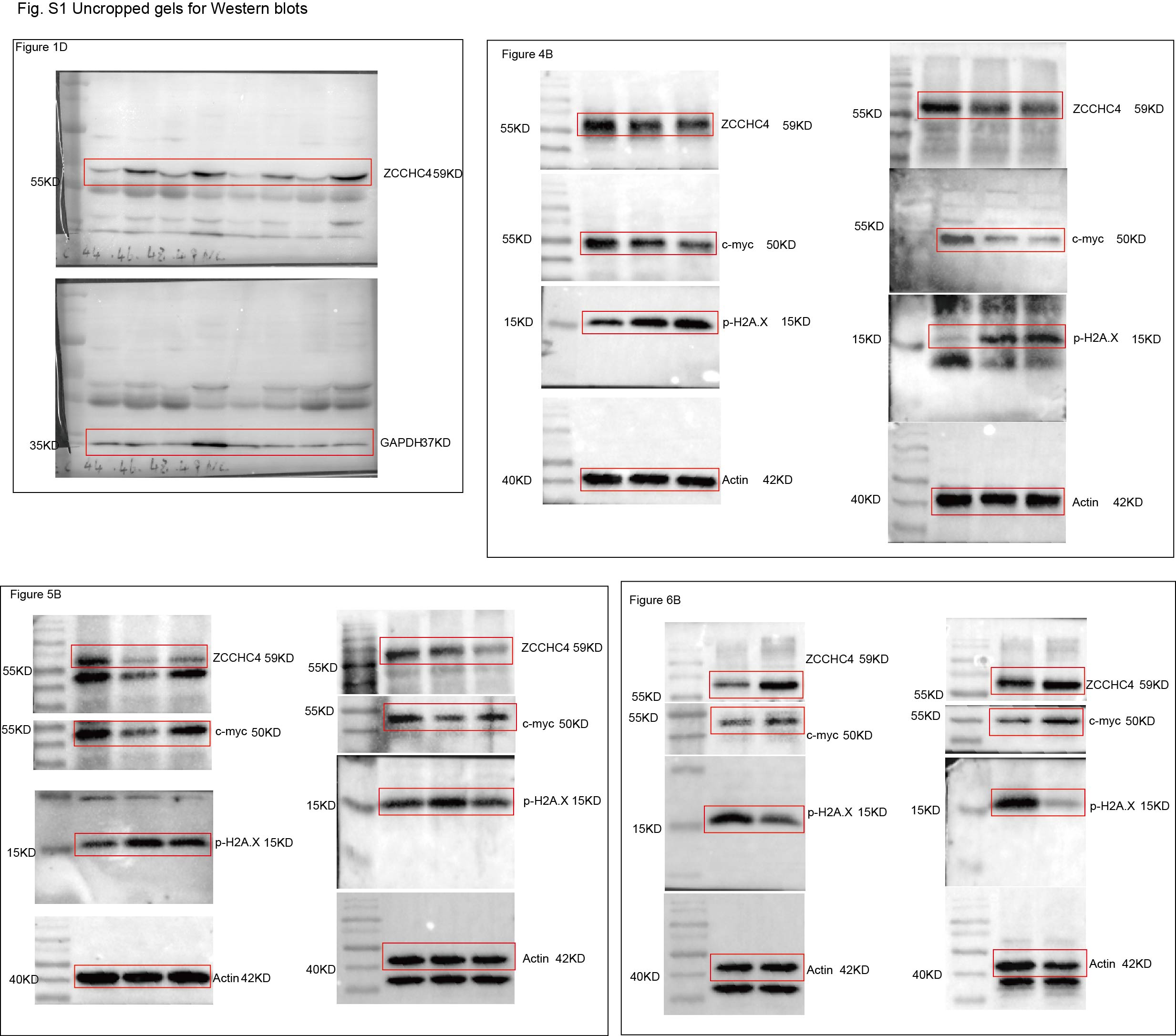

Supplement: Supplementary file 1 — Supplementary Material 1 [file 41598_2025_89628_MOESM1_ESM.jpg]
